# Coping With COVID-19: Challenges and Resilience

**DOI:** 10.1093/geroni/igab046.1206

**Published:** 2021-12-17

**Authors:** Lauren Mitchell, Lauren Mitchell, Daniel Mroczek

## Abstract

COVID-19 has introduced unprecedented challenges for older adults. At the same time, older adults have adapted to meet the challenges of the pandemic. In this symposium, we explore a number of difficulties brought about by COVID-19, while also investigating the ways in which individual, social, and community resources and strengths have bolstered older adults' resilience through the pandemic. Paper 1 investigates family caregivers of older adults with dementia living in long-term residential care facilities, a group that has been especially heavily affected by the pandemic. Using longitudinal data spanning Fall 2017-Spring 2021, the authors estimate caregivers' trajectories of well-being pre-and-post pandemic. With an exceptionally large qualitative data sample, Paper 2 examines the influence of COVID-19 on older adults' neighborhood engagement. Thematic analysis has revealed diverse patterns of response to the pandemic, as well as community and personal characteristics that have facilitated older adults' coping and resilience. Papers 3 and 4 examine how older adults' personality traits may influence their responses and adjustment to the pandemic, each using assessments of personality taken before the pandemic. Specifically, Paper 3 investigates the relationship between Big Five personality traits and older adults' concerns, precautions, and preparatory behaviors. Paper 4 explores how optimism predicts older adults' emotional responses, including worry, loneliness, and benefit-finding. Using a variety of methods and populations, these studies illustrate the challenges that older adults and their families have faced over the past year, as well key factors at the level of individuals, relationships, and communities that have contributed to their resilience.

